# Charting the Characteristics of Public Health Approaches to Preventing Violence in Local Communities: A Scoping Review of Operationalised Interventions

**DOI:** 10.3390/ijerph21101321

**Published:** 2024-10-04

**Authors:** Peter John Mennear, Alison Hurst, Katrina Mary Wyatt

**Affiliations:** 1 Gloucestershire Hospitals NHS Foundation Trust, Gloucester GL53 7AN, UK; 2 Relational Health Group, Department of Health and Community Sciences, Faculty of Health and Life Sciences, University of Exeter Medical School, Exeter EX1 2LU, UK; a.j.hurst@exeter.ac.uk (A.H.); k.m.wyatt@exeter.ac.uk (K.M.W.); 3 National Institute for Health Research Applied Research Collaboration South West Peninsula (PenARC), University of Exeter Medical School, University of Exeter, Exeter EX1 2LU, UK

**Keywords:** public health approach, violence, characteristics, realist informed, community

## Abstract

Interpersonal violence is a global driver of significant physical and mental ill health. Violence prevention is now a public health priority, and there have been international calls for the development of public health approaches to address this problem. This systematic scoping review identifies the scope of the literature and characteristics of operationalised public health approaches to prevent violence in communities. Synthesising what is meant by a public health approach to violence and the characteristics of operationalised approaches will assist future intervention development. Systematic searches of published sources (published following the World Health Assembly (WHA) declaration of violence as a public problem, June 1996 to April 2023 inclusive) were completed across six leading databases. For each identified approach, and reflecting a realist-informed methodology, data were extracted under the themes of major drivers, values and principles, key components, and community involvement. Of the 43 included studies, most were conducted in high-income countries and focussed on preventing weapon-related and youth violence. The studies from middle- and low-income countries also included responses to varying sexual and gender-based violence. There is a wide variety of identified characteristics, reflecting the diversity of violent behaviours public health approaches aim to impact. Approaches included focusing on changing norms and stopping violence at the individual level, to attempts to influence wider structural prevention opportunities.

## 1. Introduction

The prevention of violence is now well established as a global public health priority. Violence of all types is an important driver of global injury and death; homicide claims 475,000 lives each year, with many more individuals and communities exposed to physical, sexual, psychological, and economic violence-related harms [1].

Within certain populations, violence can be a leading cause of death; for example, in the United States of America (USA), homicide is the third leading cause of death for 15–34-year-olds and the leading cause for Black males of the same age group [2]. This also indicates the frequent way in which violence has uneven impacts and is a driver of inequality across and within communities. 

The declaration of violence as a leading public health problem by the 1996 World Health Assembly (WHA) gave global prominence to the development of public health interventions in this field [3]. More recently, the integration of violence prevention within the context of the Sustainable Development Goals [4] (particularly, but not limited to, the goals focused on gender equality, and peace and justice) firmly positions violence as requiring additional focus beyond the traditional methods of law and order. The World Health Organisation (WHO) leads ongoing programmes to support ongoing global public health efforts with particular areas of focus including violence against women [5]. 

Public health is concerned with problems that affect the health of populations, and aims to provide the maximum benefit for the greatest number of people through the adoption of preventative approaches and addressing root causes [6]. The inaugural World Report on Violence and Health that resulted from the 1996 declaration described the key features of a public health approach: (1) systematic data collection and epidemiological understanding of the scope and characteristics of the problem; (2) understanding associated risk and protective factors; (3) development of preventative interventions; (4) disseminating and implementing promising interventions [6]. These features therefore form the framework for interventions identified in this review. 

Risk and protective factors can be considered within the concept of the socioecological model (SEM), which describes their operation at different levels (individual, relationship, community, and societal) and how these interactions influence violent behaviours [6,7]. Therefore, public health approaches require an understanding of the complex proximate and distal causes of violence, its varied nature, and the diverse settings in which it takes place. They inherently involve multiple stakeholders and allow for greater focus on more entrenched and challenging social problems. 

The WHO typology of violence encompasses self-directed (suicide and self-abuse), collective (social, political, and economic violence such as that operating at the intra- or interstate level in order to advance specific agendas), and interpersonal violence (taking place within families or local communities, involving members of families, acquaintances, or strangers) [6]. This scoping review focuses on public health approaches that aim to prevent interpersonal violence within families and communities and includes global examples published since the WHA’s declaration. 

An initial review of the literature indicated a range of papers that either called for a public health approach to violence, describe a public health approach in generic terms, or study the implementation in a specific context [8].

International approaches include a variety of programmes in the USA and the development of national structures within the Centers for Disease Control, including activities that predate the WHA declaration [9]. Within the United Kingdom (UK), notable approaches include Scotland’s Violence Reduction Unit (VRU) [10]. In England, the government has encouraged the development of VRUs and explicitly called for a focus on prevention within ‘whole system approaches’; this was most recently through the Serious Violence Duty on relevant local public services to work together to develop joint plans to prevent violence (via the Police, Crime, Sentencing, and Courts Act 2022) [11].

As public health approaches take into account the individual, their relationships, the systems they live and work in, and the wider sociocultural, political, and economic ecosystem, realist approaches are helpful to identify the relationships between these levels and the contexts that support or inhibit health. Hence, this review takes a realist-informed approach to understand these relationships and the complex antecedents and proximal factors that influence violent behaviours and situations. Realist approaches to evidence review and synthesis embrace this complexity and enable a focus on the broader determinants of violence, in addition to consideration of the wider political and community context of local decision making. 

In realist methods, there is a particular focus on the interaction between Context, Mechanism and subsequent Outcomes (the CMO configuration) [12]. As a scoping review, there is limited capacity for a detailed realist analysis, as it does not attempt to formally identify outcomes or ‘what works’. However, by attempting to identify the types and variety of interventions that exist, it may highlight avenues for further research and prompt new thinking in practitioners. Given the topic area, the role of the community in developing and implementing public health approaches may be particularly beneficial and in line with realist principles. 

Bringing together a synthesis of what is meant by a public health approach to violence and the characteristics of operationalised interventions will inform the evidence base and the development of future interventions. 

The aim of this study was to systematically identify and describe the available published literature on public health approaches to preventing violence where these have been implemented. Recognising the complexity of the topic and variety of operationalised interventions taking a public health approach in diverse populations, this review takes a realist-informed approach [12], the aim of which is to identify the key features of these interventions but also hypothesised mechanisms and contexts that generate outcomes.

## 2. Materials and Methods

### 2.1. Methodology

In order to map and identify these characteristics, a scoping review methodology was adopted. The approach was informed by the Joanna Briggs Institute methodology for scoping reviews, which is in turn based on the Arksey and O’Malley framework as adapted by Levac et al. [13,14]. A research protocol was first developed by P.J.M in order to refine the approach, and the objectives, inclusion criteria, and methods of analysis for this review were specified in advance. Low-risk ethics approval was secured. Supervision and additional reviewer support were kindly provided by K.M.W and A.H.

### 2.2. Inclusion Criteria

The Participants, Concept, Context approach informed development of the following inclusion criteria:

- Participants—Public health and partner agencies operating at the sub-national level, and populations identified at risk of interpersonal violence.
- Concept—The characteristics of multi-agency, operationalised interventions described as following a public health approach to prevent/address/control such violence (what values and principles drive these and what their components are).
- Context—Included interventions were operationalised in sub-national geographies/areas; are not limited to a specific region of the world; and address interpersonal types of violence including physical, sexual, psychological, and neglect, where these were included in a self-described public health approach to violence.

Studies examining collective or self-directed forms of violence (as defined above) were excluded. Any studies where the focus was on online communities were excluded, as were interventions led by single agencies. 

All study types and methods were included. Due to the resources and timescale available, grey literature available from sources other than the named databases was not searched.

### 2.3. Databases and Searches

Following an iterative process, initial searches of databases including MedLine and Applied Social Sciences Index & Abstracts (ASSIA) were used to formulate the strategy and refine search terms. Following testing and consultation amongst the authors, the following terms were used to search the title and abstract fields: (public health approach*) AND (violen*). Studies were included if full texts were available, were published between 1 June 1996 and 30 April 2023, and available in the English language. 

Detailed searches were completed on the following six databases: Cochrane Library, Trip PRO, OVID MedLine, APA PsycInfo, ASSIA, and Public Health Database ProQuest. Detailed strategies for each database are included in Appendix A. Search results were imported into EndNote 20 for deduplication. A two-stage process was adopted to identify included studies. Rayyan was used to screen titles and abstracts by the lead reviewer, and full texts were reviewed using Covidence (Rayyan and Covidence are online platforms for managing systematic research). For both stages, 10% of the sample was checked by A.H. to reduce reviewer bias, and any disagreements were resolved following discussion.

### 2.4. Data Extraction and Synthesis

A data extraction form was developed within Covidence, informed by the inclusion criteria, agreed with each author, and kept under review. The fields were grouped into the following themes: descriptive information (including type of study, location of intervention); participants (including target at-risk populations, constituent membership of interventions); how approaches were conceptualised and operationalised (including influences on approach, key components); contextual (including type of violence targeted). This was analysed to identify the scope of the literature and characteristics of the approaches. These were then categorised into the following four main themes, which were decided in advance in accordance with the aims of this review: major influences driving the establishment of interventions (Context), the values and principles informing interventions, the key components of interventions, and community involvement and engagement (Mechanisms). 

## 3. Results

### 3.1. Description of Studies

**Figure 1:**
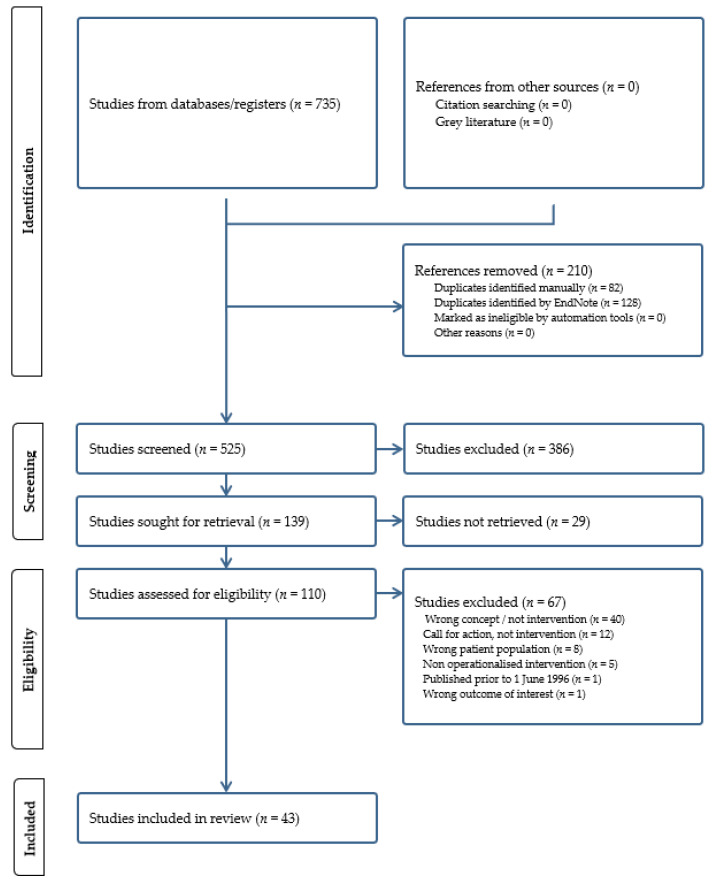
PRISMA flow diagram for the scoping review process.

The database search returned 735 studies. From these, 210 duplicates were removed, leaving 525 studies for screening by title and abstract using the inclusion criteria. Following this stage, 386 studies were excluded and a remaining 139 were sought for retrieval. Of the 110 full texts available, 43 met the inclusion criteria, see Figure 1.

**Figure 2:**
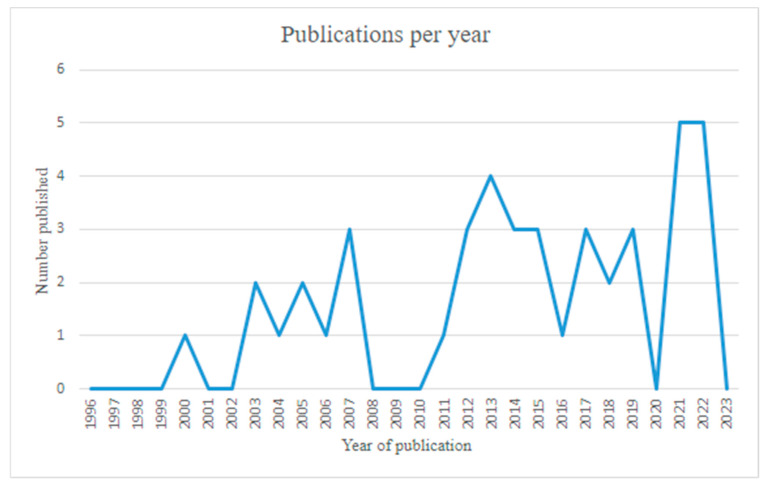
Publication dates of included studies.

Included studies comprised systematic reviews (*n =* 1), review summaries (*n =* 17), primary research (qualitative *n =* 3; quantitative *n =* 7; mixed methods *n =* 2), case studies (*n =* 7), commentaries (*n =* 5), and an official publication (*n =* 1). Publication dates were as follows: prior to 2000 (*n =* 0), 2000–2009 (*n =* 10), 2010–2019 (*n =* 23), and 2020–2023 (*n =* 10). For more details see Figure 2.

The majority of the literature focused on interventions that have been developed in high-income countries (*n =* 36), with relatively few in lower middle-income (LMIC) (*n =* 2), upper middle-income (*n =* 2), and low-income (*n =* 1) settings. Two studies included examples from countries in various income groups, one of which focused on communities within lower-income countries but also their diaspora living in higher-income states [15]. 

Some studies included reference to approaches in more than one country; those including an example from the USA were most frequent (*n =* 30). Studies also covered the UK (*n =* 7), South Africa (*n =* 2) Colombia (*n =* 1), Germany (*n =* 1), India (*n =* 1), Mongolia (*n =* 1), Uganda (*n =* 1), and Zambia (*n =* 1). A systematic review of interventions on female genital mutilation focused on the Arab League and its diaspora: Sudan, Egypt, Arizona, immigrant communities in European Union (EU) countries (Italy, the Netherland, Portugal, Spain, and the UK), Guinea, Kenya, Somalia, Iraqi Kurdistan, and Sweden. 

Given the variety in scale of interventions, the geographical level of each approach cannot always be cleanly delineated; however, a broad overview can be discerned as follows: regional including USA state (*n =* 9), city/county (*n =* 24), community (*n =* 17), and organisation (*n =* 2). On a similar basis, interventions may address more than one type; the numbers of studies that included reference to a type were as follows: use of weapons including guns (*n =* 18), youth/gang (*n =* 12), intimate partner/domestic violence (IPV) (*n =* 7), sexual (*n =* 5), child abuse/sexual (*n =* 7), gender based/female genital mutilation (FGM) (*n =* 1), and other physical including influence of alcohol (*n =* 8). 

Some studies clearly focus on provision for certain target at-risk populations including both potential victims and perpetrators: children (*n =* 9), adolescents and young adults (*n =* 19), women (*n =* 2), those at risk of IPV (*n =* 3), nighttime economy users (*n =* 2), sex workers (*n =* 1), and young Black and Hispanic (*n =* 1).

It is important to note the type and content of the literature was varied, with studies ranging from in-depth evaluation of specific interventions to surveys of the field that included at least some detail on several approaches. Due to the varied nature of this evidence base, the following thematic analysis therefore relies on the more detailed studies.

### 3.2. Identified Characteristics

**Figure 3:**
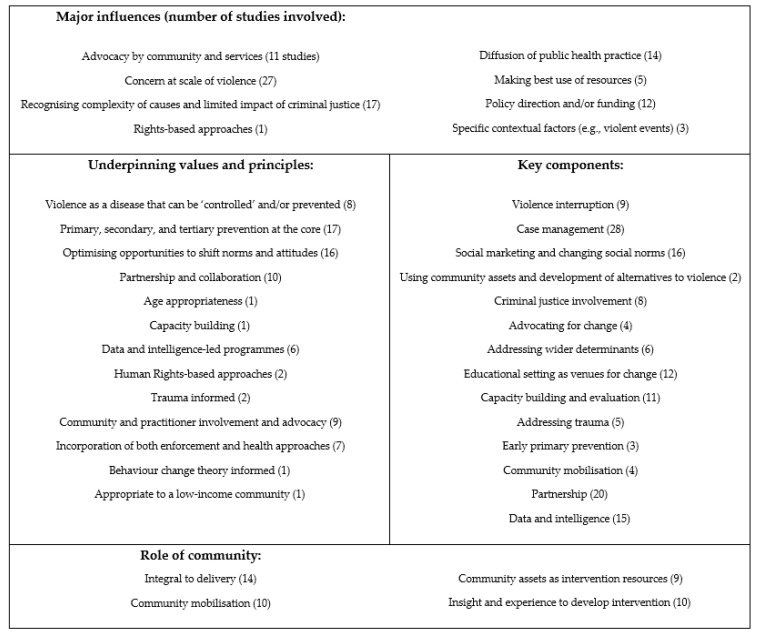
Summary of characteristics identified per review theme.

The key characteristics of the interventions have been identified and grouped thematically, and are summarised in Figure 3, with a detailed overview in Table 1, Table 2, Table 3 and Table 4. 

### 3.3. Major Influences of Interventions

For the majority of violence types, concern at the scale and impact of violence [2,7,16,17,18,19,20,21,22,23,24,25,26,27,28,29,30,31,32,33,34,35] is a principle driver of interventions, with specific concern at homicides [17,23,26,29,36,37], the social acceptability of violent behaviours [20,22,30,36], and the unequal impact of violence particularly within gender and ethnicity [2,28,35,37].

Providing a wider context for some interventions is the increasing understanding of complex root causes [2,18,21,22,25] (particularly for youth and weapon-related violence) and the adoption of the preventative approaches within public health for a broader range of violence types including IPV [9,21,28,29,38,39]. Across violence types, there is an appreciation of the limitations of criminal justice approaches alone [9,17,18,36,39,40,41], and a desire to seek improvements to how existing services are arranged [26,28,33,42,43], including desire for improved use of resources [25,29,32] and the results of advocacy amongst health workers [21,26,32,42,44]. The availability of specific funding streams was important for a minority [21,27,33,36,44,45,46]. A limited number of studies addressed context-specific drivers including the use of community-based health workers in lower-income countries [47] and a human rights approach to protect sex workers [48].

Table 1 describes the major influences which resulted in the development of a public health approach to address the issue.

**Table 1:** Identified major influences that prompted the development of public health approaches.

Major Influences Driving Interventions
**Concern at the scale and impact of violence:** - High rates of gun/weapon violence [2,16,17,18,20,21,22,24,25,27,28,29,32,35], youth violence [19,23,26], intimate partner violence (IPV) [30,33], child sexual exploitation (CSE) [7], alcohol [34], fixated threats [31] - Concern over homicide as key driver [17,23,29,36,37], particularly within Black/minority groups [26] - Community and social costs [30,34], economic costs [21,34,37] - Outcomes on victims of IPV [30] - Prevalent community norms including acceptability of IPV [30], violence/weapon carrying [20,22,36] - Future impact of exposure [26] - Gang-related retaliations [49] and territoriality [22] - School shootings in the United States of America (USA) [50] - Disparities in impact—ethnicity and gender and disadvantage communities [2,28,35,37] **Growing understanding of varying/complex influences on violent behaviours:** - Structural/internalised racism [28] - Recognition of complex root causes [2,18,21,22,25] and factors operating across the socioecological model (SEM) [23,43,49,51] - IPV closely linked to male dominance in society and reflective of inequalities [30,40] **Increased adoption of public health approach/theory:** - Influence of public health approaches including World Health Organisation (WHO) calls for action [9,21,28,29,38,39] - Primary prevention as best evidenced (e.g., parental programmes), but action throughout life course and levels recognised to be necessary [49,51], evidence highlighting the potential to break cycles of violence [26] - Examining targeted and universal applications of empirically supported violence prevention in school teaching [46] - Increasing interest in trauma informed approaches including the impact of exposure to violence and future victimisation and perpetration [21,28] - Influence of USA community initiative models but tailored to local area [18,22] - Importance of addressing gaps in evidence base [29,30,45,46] **Advocacy by community:** - Community-led commission/advocacy [2,35] **Advocacy of service providers/improvements to healthcare:** - Healthcare provider-led advocacy and recognition of role in anti-violence [21,26,32,42,44] - Desire to improve post-event follow up and treat non-physical injuries [26,28,42] - Recognition law enforcement data is incomplete versus Emergency Department (ED) health records on violence attendances [34,52] - Systematic barriers to safety, permanent sense of acute crisis, lack of trust and safety for both staff and patients in healthcare, defensiveness [28] - Development of integrated hospital-based intervention approach after separate sexual and domestic violence teams [27] **Desire for improved use of resources and partnerships:** - Leading consumer of healthcare resources (compared to other issues) [32] - Desire for strategic collaboration and response [25] - Desire to bring together existing programmes [29] - Refinement/development of programmes already in existence [33,43] **Limited or negative impacts of criminal justice approaches:** - Law enforcement limited long term impact alone and not sustainable [17,18,36,39], a reactive law enforcement approach to IPV [9,40,41] - Iatrogenic effects of incarceration [39] **Policy direction and/or funding:** - Availability of funding [21,27,33,36,44,45,46] - National guidance and policy/call for action [23,34,43] - Legal obligations introduced at USA state level [40] - National development of research and potential of preventive interventions [7,45] **Context specific drivers/delivery mechanisms:** - Response to pressure on civil society following apartheid [29] - Response to specific events, e.g., community/police tensions [21] - Use of community health workers as one of limited options in lower middle-income countries (LMICs)—resources intensive models not transferable [47] **Examples for specific types of violence—violence against sex workers:** **Influence of rights-based approaches:** - Human rights and violence prevention intervention to focus on violence prevention, response, and/or police treatment of sex workers [48]

### 3.4. Values and Principles

Broadly speaking, the values and principles informing interventions can be grouped into two main concepts: (1) the concept of violence as a communicable disease that can be ‘controlled’ in a similar fashion, with the emphasis on individual exposure and actions [17,18,20,21,24,38,49,53], and (2) a greater focus on the complex causes of violence and upstream preventative interventions [16,18,21,22,23,24,25,29,36,42]. 

Further important principles include focusing on opportunities to change norms and attitudes challenging the acceptability and also the inevitability of violence [17,19,30,36,46,51,53,54], the role of collaboration to both develop and sustain interventions [2,18,20,22,25,27,35,37,44,53], as well as the influence and use of data to target limited resources and drive improvements [2,25,34,37,44]. An important theme within some studies that focused on the mental health effects of trauma (and the cycle of violence that can result) is the use of trauma-informed approaches [26,28]. Although the literature notes the limitations of criminal justice, a balance of enforcement and health approaches can still be discerned [18,22,39,41,50], as can the principles of practitioner and community involvement [27,28,29,32,34,40,48,52]. Studies focused on IPV included a focus on the importance of age appropriateness [43] and using behavioural change theories to influence both activities and the planning stages of interventions [30]. 

**Table 2:** Detailed summary of values and principles underpinning the interventions.

Values/Principles Informing Interventions
**Violence as a communicable disease (CD):** - Controlling exposure and transmission through CD methods (at the individual level) [17,18,20,21,24,38,49,53] **Prevention at the core (inc. targeted/multi-levelled approaches):** - Prevention and improving health and safety at a population level [18,25,29,36] - Primary preventative approach for at-risk families/children/adolescents [7,9,33,41,43,45,46,53,54] - Promotion of positive parenting strategies/reduce negative or coercive strategies [45,46,47], and a focus on maternal mental health [47] - Adoption of secondary and tertiary prevention principles [26,51] and harm reduction approaches [31] - Influence of universal and targeted approaches [46,51] - Primary prevention of CSE—focus on adults and involvement of adults whether or not perpetrators (rather than waiting for children to disclose). Belief that abusers can stop and should be offered support, whilst being held accountable [54] **Focus on complex causes including social determinants of health:** - Address constellation of risk factors—recognise fundamental problems with relationships with other, e.g., family [30,41,43] - Informed by science, common causal factors, and early intervention [46] - Recognising complex health inequalities require integrated multi-level approach required (e.g., complex risk factors including stigma, sexually transmitted infections (STIs), unintended pregnancies, exposure to violence, human rights violations) [48] - Addressing structural and social determinants of health (including post-event) [16,21,22,23,24,29,42] **Optimising opportunities to shift norms and attitudes:** - Opportunities from focusing on post-event ‘teachable moments’ [24,26,27,42] - Prevention of re-victimisation/cycle of violence [27], promotion of alternative norms and less harmful resolutions [17,36,51], addressing the complex nature of gang memberships [49] - Address community acceptability of violence/social justice approach [19] - Addressing power dynamics and negative dominant narratives about young people [23], primary and secondary activity to address norms and stereotyping—combination of individual and community/societal levels [30,54] - Combining identification of high-risk and broader education for wider community [17] - Attempt to sensitise authorities to scale of issue [32], including IPV [9,30] - Promotion of healthy relationships [43] - Engagement of parents and educators as strong influences at age 11–14 [43] - Tackling perceptions that violence is not preventable (relating to child maltreatment) [46,53] **Role of partnership and stakeholder in the development and sustainment of interventions:** - Partnership approach (including long term approach) [2,18,20,22,25,44,53] - Benefit of bringing together multiple organisations/perspectives to identify complex causes and consequences [18,27,35,37,44] **Use of data and intelligence to target use of limited resources/innovate interventions:** - Focusing of limited enforcement and intervention activities through analysis and innovative response [2,25,37,44] - Benefit of collaboration including use of data [34] - Contextual social analysis to complement technical knowledge [29] **Incorporation of enforcement and health-focused approaches:** - Balance of enforcement and rehabilitation and support [18,22,39] - Enhancing student safety through environmental school route safety improvement/and active travel [50] - Mandatory training as an atypical use of legal framework within public health approach on IPV [40] - Reduce formal approach and limit police involvement at early stages of response [40], diversion from criminal justice approaches [31] - Complementary to law enforcement [41] **Trauma-informed approaches:** - Trauma-informed approach to address mental health consequences of violent injury, prevent re-injury, and improve life course trajectories of injured youth [28] - Addressing the cycle of violence—‘hurt people hurt people’—informed by the psychological, biological, and behavioural risk factors that derive from violence and adversity (including in childhood) [26] **Community and practitioner involvement and advocacy:** - The unique role of practitioners to witness and influence [27,29,32] - Recognition of potential role of nurses/health staff [34,52], education and support for staff [28] - Encouraging community responsibility [54] - Grounded in experiences of and advocacy by sex workers, to address legal and structural barriers [48] - Utilisation of specific contexts, e.g., a unique social context and trusted relationships (to receive sensitive information/deliver help) [40] - Combining public health (quantitative/population level) and community development approaches (organic/bottom up) [29] **Human rights based approach:** - Raising awareness of women’s human rights [30,48] **Examples for specific types of violence—intimate partner violence:** **Cognisance of age-appropriate interventions:** - Address transient relationships at this age [43] - Focus on foundational stages of development [43], acknowledge differences to other youth violence [43] **Capacity building:** - Hypothesised to lead to prevention activities (initiating or expanding prevention activities) [33] **Informed by behavioural change theory:** - Transtheoretical Model (TTM) of behaviour informed design [30] **Development of approach in low-income environment** (recognising most developed in high-income) [29]

### 3.5. Key Components of Interventions

The varied nature of the literature is demonstrated by the range of intervention components. The use of violence interruption amongst high-risk individuals originated in the Cure Violence model in Chicago and is represented in several USA-based approaches [16,17,18,19,20,21,36,38,51]. Case management and individual support encompassed outreach provision to engage and support those at highest risk in the community [16,17,18,19,20,21,22,23,24,25,36,38,49], hospital-based referrals leading to wrap-around care and referrals [2,24,26,27,28,29,42,51], and the provision of support to those at risk of future child maltreatment in the community [45,46,47,53]. Specific attention to past trauma featured within these projects [27,28,29,42]. 

Shifting norms at the community level was manifested through community programmes, events, and social marketing for interventions aimed at youth and gun and knife violence [17,18,19,20,23,24,32,36,38,46,49,51], as well as attempts to reframe IPV [30,33]. Two approaches included awareness and treatment options for child sexual exploitation [7,54]. Data and intelligence were used in order to identify at-risk individuals [2,17,18,19,21,22,25,34,36,39,46,52] and in aggregate form to inform planning of targeted interventions [34,37,52]. A unique use of data in one study focused on post-event review of homicides in order to identify future opportunities for prevention [37]. Furthermore, close links with and alternatives to criminal justice systems were incorporated into some approaches [18,22,23,35,37,39,49]. 

Education settings were a frequent base for preventative programmes, including both targeted and universal skill and awareness training in curricula [7,18,23,29,30,33,35,41,43,46,53,54]. A limited number of approaches were identified that traced violence prevention actions to the early life stage [18,39,51].

Careful consideration to development and evaluation of interventions was particularly evident in some studies [18,25,27,29,30] and capacity-building included training of staff groups and support for local public health offices [2,9,30,32,33,43,44,45,46,47,48]. The creation and focus on partnership working was a key element [2,9,17,18,21,22,25,26,27,28,29,30,33,34,35,36,37,39,42,52]. Reflecting the scope of the evidence, some approaches addressed the wider determinants of health including economic and job support [18,23,29,35,48,50,53].

**Table 3:** Detailed summary of key components of interventions.

Key Components of Interventions
**Violence interruption to prevent individual/group conflicts (at the individual level):** - Using violence interrupters to detect and interrupt conflicts, identifying and treating highest-risk people [16,17,18,19,20,21,36,38,51] **Case management and support at the individual level:** - Outreach workers—connect, challenge thinking, engage, and link high risk with positive alternative opportunities (or similar) [16,17,18,19,20,21,22,23,24,25,36,38,49] - Case workers: embedded within/link with EDs to engage with victims—intensive case management and wrap-around including referrals to community resources and peer support—may include home visit and follow-up [2,24,26,27,28,29,42,51], support for adolescents in abusive relationships [29,30] - Tiered approach to prevent child maltreatment from brief consultations to longer term depending on risk [45], multi-level parenting programmes [46], including intensive home visiting [47] and home safety and child health training [45], and support during pregnancy [53] - Development and offer of treatment to possible child sex offenders [7] - 24 hr crisis management to address violence and advocates during wrongful arrest of sex workers [48] - Maintenance of specialist case management system for fixated threats. Cases assessed and joint police/mental health assessment of appropriate health or police action [31] **Addressing individual trauma and past experiences of contact with service providers:** - Culturally competence leads to address previous negative experiences of healthcare [28] - Seek to understand issues, communication, and trauma, and address anger [27,28,42], educating staff on effects of trauma and stress, mindsets of clients, and tools to change individual and group behaviours - using SELF concepts ie. Safety, Emotions, Loss, Future [26,28,29] **Development of alternative activities and community assets:** - Creation of alternatives for communities and upstream universal approaches, responding to local risk and protective factors, use of community assets [18] - Mandatory recognition and signposting training for salon workers (not mandatory reporting) [40] **Social marketing approaches and shifting norms at community level:** - Changing behavioural and social norms through community programmes and events [17,18,20,36,38,46] - Social marketing anti-violence campaign [24,32], specific attempts to reframe IPV using media and outreach [30,33] - Promotion of ‘guiding responsibilities and expectations in adolescents’ and positive racial identities [19,23,49,51] - Media and community campaigns to raise awareness of child sexual abuse (CSE) warning signs [54], advertising of treatment for potential offenders [7], helpline for potential CSE abusers, friends and family of suspected abusers, and concerned community members [54] **Advocating for change with policy makers:** - Explicit aim to influence local and national policy makers (bottom up), including legislators [29,30,32,33] **Improved use of data and intelligence:** - Use of data to inform approach on specific conflicts and identify highest risk [2,17,18,19,21,22,25,36,39], including IPV [46], and ED statistics [34,52] - Data sharing within partnerships to inform prevention strategies [34,52] - Post-event review of homicides to inform future prevention activity [37] - Systematic data collection to inform design [30] and understand attitudes to physical abuse of children [53] **Key role for ‘partnerships’:** - Use or creation of formal/multi-agency partnerships [2,9,17,18,21,22,25,26,27,28,29,30,33,34,35,36,37,39,42,52] - Co-production within partnerships [18] **Criminal justice included in the approach:** - Potential enforcement as integral part of approach [18,22,37,39,49]/targeting of hot-spots [18] - Emphasis on voluntary participation and collective gang responsibility—self-referral and behaviour contracts (with no incentive aside from offer of support) [18,22] - Reducing school based arrests and diversion from criminal justice [35], incorporation of restorative justice [23] - Skill-building and legal empowerment for sex workers [48] **Wider determinants component:** - Environmental improvements, e.g., improved physical conditions on school routes, safer pathway, police presence on travel routes [18,35,50], and clean and green initiatives [23] - Economic and social development—community and small business partnerships seen as primary prevention [29], job creation and skills [35,53], parental support, health and wellness, behavioural/mental health, basic needs, and food insecurity [35] **Primary prevention early in life course:** - Primary prevention activities within antenatal/childhood/families (e.g., enhanced home visiting) [18,39] - School referrals for disruptive pupils at early elementary school—parental training (positive reinforcement, effective punishments, and monitoring) and pro-social skills for children [51] **Educational setting as a hub for interventions:** - Universal school-based programmes focusing on social and emotional development [18,29] and targeted interventions for high-risk youth in school curricula. Universal interventions for all students at grade level/risk screening in school health centres [23,46] - Continuum of support for children, young people, and families: after/summer school activities, school enrichment [35,53] - After school projects and alternative suspension programmes and academic support/counselling for suspended pupils [35] - School-based programmes for IPV for 13–15-year-olds—changing norms regarding dating/gender stereotyping/conflict management/bystander strategies [30,33,41,43,54]; training for parents and teachers [43] - Education and training for university students about risk factors for sexual abuse, empowering peer interventions, and encouraging reporting. Aimed at reducing victimisation, as well as how to talk to someone who may be at risk of harming someone or being harmed [7] **Development and evaluation of interventions:** - Pre-intervention—significant investment in engagement with internal and partner support and buy in, and community resource network [27,29] - Ongoing development of interventions [18,25] staged approach to develop, build networks, consolidate over time [30] **Community mobilisation:** - Significant community engagement in planning of intervention [29] - Community mobilisation/dialogue [23,30,49] - Faith-based leader involvement [49] **Capacity building:** - Creation of formal offices focusing on violence prevention [2,44] - Building capacity of local health departments, e.g., boost surveillance by Centers for Disease Control and Prevention (CDC) work with communities to identify indicators for teen dating IPV [9,30,33,43] - Health/cross-sector provider training [30,32,33,45,46], including for child abuse [47], violence against sex workers, and police training on sex work/STIs and legal position [48]

The identified systematic review on female genital mutilation [15] is included in the descriptive data but as the individual studies included in the systematic review were not identified in the search for this scoping review, their characteristics are not included in Table 1, Table 2 and Table 3. In summary however, the characteristics of the results of those studies align with those in Figure 3, in particular, the influence of preventative methods, commitment to human rights, and a strong focus on challenging social norms whilst being respectful of the prevailing culture, including the use of a variety of educative and marketing techniques, training of provider staff, and community representation. Multi-sectoral action that targeted norms operating at several levels appeared most effective.

### 3.6. Role of Community Engagement and Involvement

Communities provide both the setting for, and assets that can contribute to the development of, public health approaches to preventing and addressing violence. The key identified characteristics regarding their role in delivering interventions are detailed in Table 4.

**Table 4:** Summary of community involvement and engagement.

Presence/Method of Community Engagement
**Community members integral to delivery:** - Community members run interventions and/or train health workers on violence reduction [20,21,29,38] - Violence Interrupters/Outreach workers live in the same communities (may have violent histories/prison experience). Used due to having the ‘street knowledge’ that ‘cannot be taught’. Credible messengers and un-judgemental [17,20,21,24,36,38], youth [23,51] - Influential older youth to act as ‘brand ambassadors’/use of recognised older authority figures in communities [30,43] - Blue Ribbon Commission leading intervention as a grassroots organisation [35] - Community consultation to listen and review and recommend on actions to prevent homicides [37] - Community resilience teams formed by neighbourhoods to increase engagement, cohesion, and resilience in response to chronic violence [16] **Community mobilisation an integral part of intervention:** - Community mobilisation and events including rallies, marches, and barbecues to propagate anti-violence messages and positive relationships with police and politicians [17,18,21,29,38]; training and social marketing within IPV responses [30,33]; community coalition building—building of unified no violence messaging and immediate response when violence occurs [17,18] and in response to youth violence [23,49] - Empowerment through community advocacy groups [48] **Community assets as a source of referral and support:** - Community agencies and resources as a source of referral/support/assets [18,21,22,27,29,42], including IPV [30,40] and violence against sex workers [48] - Use of culturally diverse locations to deliver IPV intervention [40] **Insight an explicit part of intervention development:** - Community (individuals/groups) as a source of understanding and context building in development of intervention [18,27,29] including IPV response [9,30], developing collaborations to ensure cultural and context of youth violence response is appropriate [19,23,26]; ensure IPV response socio-culturally relevant to ensure communities can make surface adaptations (without impacting on effectiveness) [43] - Focus on ethical protections for use of data (given minority communities’ experience of structural racism in this arena) [2] - Importance of continuous negotiations between formal and organic indigenous knowledges; managing leadership tensions and vested interests in changing communities; nurturing of partnerships [29]

Although not universally present in all studies, there are four groups of characteristics relating to community engagement and involvement: (1) interventions with community members being integral to delivery including their role as peers with lived experience and significant community and lived experience [16,17,20,21,23,24,29,30,35,36,37,38,43,51], (2) community mobilisation and empowerment [17,18,21,23,29,30,33,38,48,49], (3) the use of community assets as a source of referral and support [18,21,22,27,29,30,40,42,48], and (4) community insight explicitly used in intervention development [2,9,18,19,23,26,27,29,30,43]. 

### 3.7. Intervention Outcomes 

As a scoping review, this study cannot assess the effectiveness of interventions, and overall, the literature is relatively limited in terms of identified outcomes. However, some broad themes can be determined where outcome information is available. Identified outcomes include quantitative reductions in rates of violence and other costs [20,34,39,42,48,51,52], influencing wider service delivery such as return to community-focused policing [16], and more subjective improvements to working relationships and commitments from partners [35,37], which it was envisaged would lead to reductions in violence that had not yet been realised. Where levels of joint engagement have not been sustained, interventions may be discontinued or face obstacles [18].

The Cure Violence model has been implemented in several USA neighbourhoods with mixed outcomes across Chicago, Baltimore, and Phoenix, appearing most suited to small geographic areas with high gun violence [18], with the Department of Justice rating the intervention as ‘promising’ rather than ‘effective’ [17,36,38]. 

Where evaluation has taken place, challenges have been identified due to confounding and blurring of the geographical definition of interventions, the contiguous nature of communities only partly covered by interventions [17], difficulty maintaining long-term fidelity to approaches in the context of changing political priorities [17], and the possible effect of concurrent policing strategies [18,22]. 

### 3.8. Variety of Public Health Approaches to Addressing and Preventing Violence

**Figure 4:**
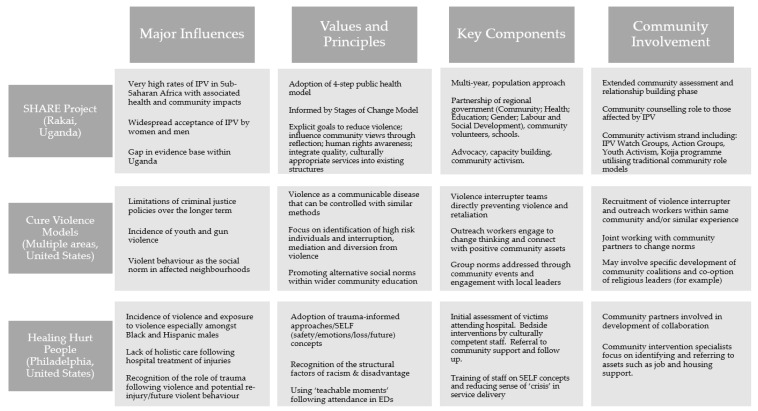
Case studies of three varying approaches.

To demonstrate the variety of approaches, three case studies are highlighted in Figure 4: a multi-level IPV approach in a low-income country; a summary of the violence interruption and outreach-focused Cure Violence programme; and a hospital patient trauma-informed project in a high-income country. 

The SHARE project is an example of a multi-partner regional programme of activities to address the specific issue of IPV by shifting community norms and provision of support to those affected by IPV, with a strong emphasis on understanding and involving communities in the development of the project [30]. Cure Violence is situated within a high-income country and seeks to change social norms in identified local communities but with a greater focus on preventing, or ‘interrupting’, violence within the immediate time period during which it is perceived as being likely to occur [16,17,18,19,20,21,36,38,49,51]. By contrast, the Healing Hurt People approach focuses on the period after violence has taken place and attempts to use the opportunity following hospital admission of victims to intervene and provide holistic support packages, with the aim of preventing secondary and tertiary harms [26,28].

Novel approaches can be seen across the identified evidence base; in Milwaukee, an interagency collaboration on homicide uses a post-event review by both practitioners and community members once relevant information can be released, and this enables identification of potential opportunities for future prevention, allowing for recommendations to be made to senior decision makers. Early outcomes demonstrated improved joint working and provision of translated education resources [37].

The full impact of the COVID-19 pandemic on global public health continues to be understood. Within the evidence base some early themes in relation to violence during the pandemic can be identified. Concern at the perceived increase in violence in the USA during the pandemic was highlighted in contemporaneous data and the manner in which the pandemic highlighted inequalities in society that intersected with the disproportionate impact of violence on some communities, including ethnic minorities [20,23]. The manner in which violence had increased at the same time as other epidemics including Ebola in the Congo was also noted [20]. COVID-19 adaptations to programme delivery were identified in hospital-based interventions [27]. However, the role of the pandemic did not feature substantially within the data, particularly in terms of how interventions were constructed and implemented, and therefore future reviews may wish to consider this further.

## 4. Discussion

This review demonstrates that public health approaches to violence comprise a wide range of characteristics. This variety may be partly explained by the diversity of violent behaviours that are now targeted through a public health lens; although the evidence remains focused on addressing weapons/youth violence, preventative approaches are being extended to types of violence more usually dealt with via justice systems, for example, child sexual abuse [7,54]. 

Overall, conceptualisation of public health approaches can broadly be grouped into those focusing on how prevention can be actioned at the individual level (which can lead to a focus on those ‘at-risk’ or already subject to the impact of violence), and those that focus on multiple levels of the socioecological framework and broader structural factors. Although the former does not preclude activities operating at the community level (particularly with regard to challenging the acceptability of violence) and the latter can encompass support at the individual level, the multi-level approach implies a wider range of action of upstream activities. Individual level actions were largely focused on secondary and tertiary prevention, although there were examples of targeted primary prevention approaches including work with families and in the early years [18,39]. 

The small number of studies from low-/low middle-income countries cover the range of violence types, although they are driven by some unique contexts (including the role of non-governmental organisations in post-apartheid South Africa) and how community health services can be delivered when resources are scarce [47], and include the only studies to particularly focus on human rights influences on gender/intimate partner violence [30,48]. 

However, the literature is overwhelmingly weighted towards the perspective of approaches based in high-income countries, the USA in particular. This may be reflective of the long-standing interest in violence within the United States public health community. Although not exclusively, there is a particular focus on the role of Cure Violence-influenced programmes (*n =* 10 studies describe the implementation of this programme to some extent). 

By their very nature, public health approaches involve more than a single group or organisation in planning and conception, but this takes various forms. A continuum of activities can be identified whereby partnerships are (or appear to be) relatively narrow and focused on delivery (including the specialist response to fixated threats in the UK [31], to the more common use or creation of multi-agency oversight of approaches through existing or new partnerships [2,9,17,18,21,22,25,26,27,28,29,30,33,34,35,36,37,39,42,52], through to a focus on co-production of strategies and formation of joint teams and co-location [18]). 

Partnerships were not always natural formations of locally based organisations. In Baltimore’s implementation of the Cure Violence approach, certain neighbourhoods were selected on level of violence rather than community capacity, and local delivery organisations were recruited through open competition, and therefore had not necessarily worked in those areas [36]. It is also important to recognise that although public health approaches involve a move away from law enforcement alone, the police and similar agencies frequently remain involved in their delivery [18,22,37,39,49] and indeed can lead such initiatives [18]. Another aspect is the way public health techniques, in particular the focus on using data and intelligence to inform actions, are utilised to improve traditional policing techniques (such as crime prevention and detection activity) [34]. 

The full involvement of the community in violence prevention has previously been identified as a crucial element to drive a sense of ownership of the issue and solutions [46]. This review found a similar common thread of how the context of the individual and their community is fundamental to the public health approach and involves stepping away from purely reactive approaches. The role of communities varied from multi-year involvement in consultation efforts [29,30] to being integral elements of operationalised approaches, including recruitment of community members in service delivery [20,21,29,38] or key roles in the oversight of local work [35]. The role of community opinion and efforts to change local norms regarding the use and acceptability of violence was a key element of many of the studies. It is also interesting to note that Wilmington’s Blue Ribbon Commission into youth violence was community-led and focused on the broader determinants of health; it provided an extensive range of educational, economic, and community support, including addressing of basic food needs as ways to reduce potential drivers of violence [35]. 

It can be hypothesised that community and cultural insight and appropriateness are the overarching mechanisms that drive outcomes in a significant proportion of the evidence base identified in this review. The very personal nature of the impact of violence reinforces the importance of seeking to truly understand community contexts, and importantly, the non-physical impacts of violence, both on victims and the wider community [2,21,26,28,30,34,35,37]. This is apparent most clearly in those approaches that specifically seek to address the impact of past trauma and engage with victims on this basis, in addition to training service providers, including healthcare providers, to be more mindful of the impact of prior stressors and adverse experiences [26,27,28,29,42]. 

Within England, developing public health approaches to violence are advised to follow the ‘5C’ formula [18]; this means close attention to Collaboration, Co-production, Co-operation [in intelligence sharing], Counter-narrative, and Community consensus. The evidence identified in this review, although varied, appears to align with much of this approach. Taking this further, a Human Learning System approach is one method by which the complexity of individual lives is more fully appreciated within public service design; this has been used to commission homelessness services and support organisations to better understand the mindsets and relationships experienced by the most underserved sections of society, and may be a fruitful way of exploring future approaches to violence [55,56]. 

The research base would benefit from additional coverage beyond high-income countries to explore the characteristics of ‘public health approaches’ in lower-income countries. Although not exclusively, middle- and low-income countries can be heavily impacted by particular types of violence, including the impact of homicide on young people in Central America [57]. 

The relatively limited identification of outcome evaluations is consistent with a World Health Organisation (WHO)-led global Delphi study (2017) that found the need for greater evaluation of interpersonal violence prevention projects before widespread adoption of preventative measures could be recommended [58]. The diverse nature of settings and approaches and the difficulty in evaluation may lend weight to using realist approaches during future research into this topic. This will allow for a greater range of information to be synthesised and particularly focus on the challenging environments in which approaches to violence are developed. 

Interventions to address violence are inherently political due to their sensitive nature and often immediate impact and visibility in the news cycle [59]. Political intervention (at a variety of levels) may also be the prime driver of a public health approach in a locality. This is highly unlikely to result in a formal trial and does not always contain formal evaluation; nevertheless, what are self-consciously termed ‘public health approaches’ to violence are then developed and are therefore worthy of investigation [17,18]. It is recognised that such interventions are at the mercy of subsequent politically led changes in policy; however, this only serves to highlight again the complexity of the wider environment. 

This review was not restricted by geography and therefore represents a global summary of the literature within the defined criteria. As the scope is broad in terms of geographical extent and violence type, this limits the amount of attention given to particular types. Due to time and resources, this review was restricted to published evidence available through the named databases, and did not include specific searches or categorisation of grey literature. A hand search was originally included in the protocol, but this was removed due to capacity. 

If an approach had the hallmarks of but was not described as a ‘public health approach’, it would not have been identified, and it is possible that significant elements of the research base remain unaccounted for. Researchers and practitioners interested in a wider evidence base can access the helpful Violence Info resource hosted by the WHO [1].

## 5. Conclusions

Public health approaches to the prevention of interpersonal violence continues to be of interest to global law enforcement, politicians, and public health practitioners and commissioners, and remains high on the policy agenda, including within the UK. The aim of this review was to describe the scope and characteristics of public health approaches to violence prevention and management. By drawing attention to the range of characteristics and the many driving influences, the findings may be of assistance to practitioners seeking to develop approaches in their own environments.

## Data Availability

Dataset available on request from the authors.

## Appendix A. Search Strategies

Detailed searches were completed on the following databases: Cochrane Library, Trip PRO, OVID MedLine, APA PsycInfo, ASSIA, and Public Health Database ProQuest. Detailed strategies for each database are below.

Studies were included if full texts were available, were published between 1 June 1996 and 30 April 2023, and available in the English language. The date range covers the period since the 1996 World Health Assembly declaration of violence as a leading public health problem.

**Date of Search**	**Database**	**Platform**	**Terms**	**Filters**	**Results**
10 May 2023	**Cochrane Library**	https://www.cochranelibrary.com/?contentLanguage=eng	“public health approach*”:ti,ab AND violen*:ti,ab	June 1996–April 2023	4
10 May 2023	**APA PsychoInfo**	https://ovidsp.dc1.ovid.com/ovid-a/ovidweb.cgi	Public health approach*.ti,ab AND Violen*.ti,ab	1996–2023 English Not related terms	157
10 May 2023	**Applied Social Sciences Index & Abstracts**	ProQuest.com	(ab(“public health approach??”) OR ti(“public health approach??”)) AND (ab(violen??) OR ti(violen??))	English June 1996–April 2023	58
10 May 2023	**Public Health Database**	Proquest.com	(ab(“public health approach??”) OR ti(“public health approach??”)) AND (ab(violen??) OR ti(violen??))	English June 1996–April 2023	103
10 May 2023	**Medline**	Ovid Medline	Public health approach*.ti,ab AND Violen*.ti,ab	1996–2023 English Not related terms	172
12 May 2023	**TRIP Pro**	https://www-tripdatabase-com.uoelibrary.idm.oclc.org/	(“public health approach” OR “public health approaches”) AND (violence OR violent)	(No date filter but earliest study was 2000)	241
